# Supplementary material for: T cell infiltration into the brain triggers pulmonary dysfunction in murine Cryptococcus-associated IRIS
Source: Nat Commun. 2023 Jun 28;14:3831. doi: 10.1038/s41467-023-39518-x (PMC10307837; doi:10.1038/s41467-023-39518-x)
Supplement: Supplementary file 1 — Supplementary information [file 41467_2023_39518_MOESM1_ESM.pdf]

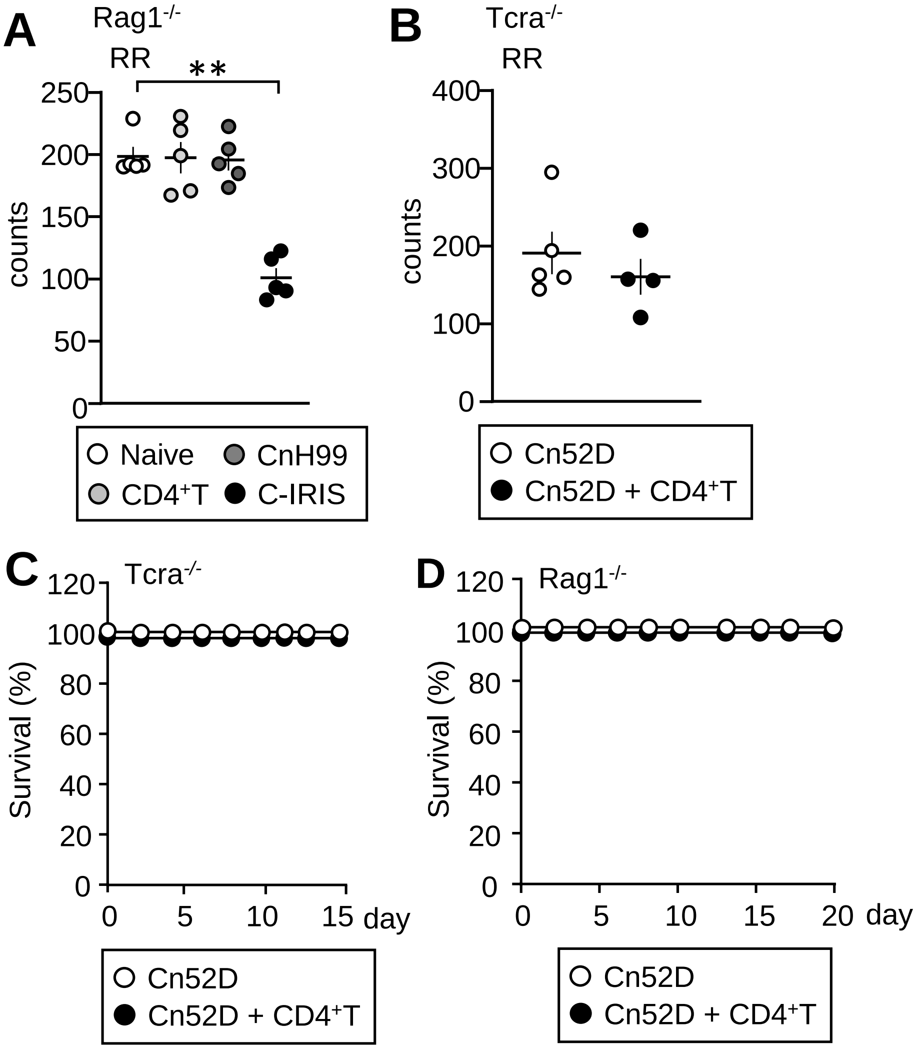

Supplementary Figure 1. Respiratory rates and survival analyses in *Rag1*$^{-/-}$ and *Tcra*$^{-/-}$ mice. (A) Respiratory rates in four cohorts: naïve *Rag1*$^{-/-}$ mice that 1) received neither CnH99 infection nor CD4^{+} T cell transfer, 2) received CD4^{+} T cells for seven days, 3) received CnH99 infection for three weeks s, and 4) received CnH99 infection for three weeks and then CD4^{+} T cells for seven days (C-IRIS). $n=5$ mice per group. Tukey's multiple comparison tests were used for statistical analyses following one-way ANOVA. **: $p < 0.01$. Data are presented as mean values ± SEM. **(B)** Respiratory rates in *Tcra*$^{-/-}$ mice that received Cn52D only or Cn52D and CD4^{+} T cells. $n=5$ mice per group. Data are presented as mean values ± SEM. **(C, D)** Mouse survival in *Tcra*$^{-/-}$ (C) and *Rag1*$^{-/-}$ (D) mice with Cn52D infection only or Cn52D and CD4^{+} T cells. $n=5$ mice per group.

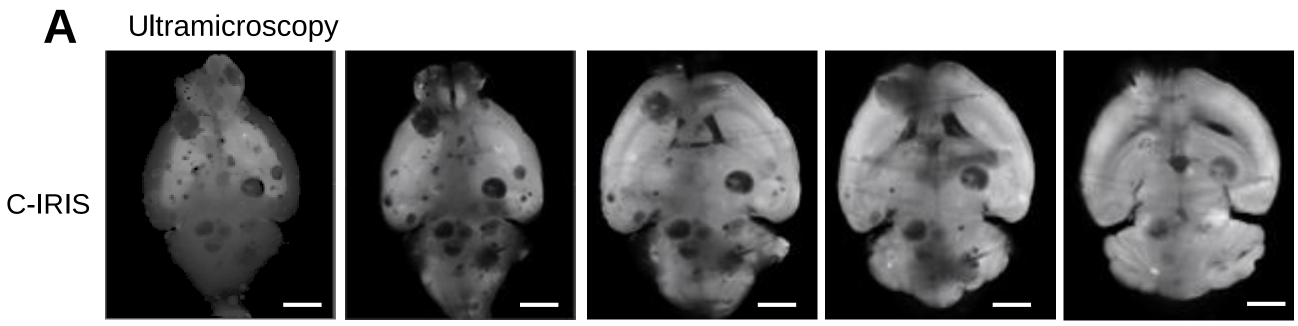

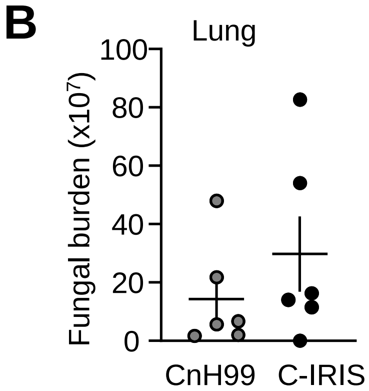

Supplementary Figure 2. Tissue examination and fungal burden. (A) Representative sequential image in one brain of C-IRIS (CnH99 pre-infection for 3 weeks plus $CD4^+$ T transfer for 7 days) *Tcra*$^{-/-}$ mouse using ultramicroscopy. The scale bar indicates 2000 µm. Experiments were repeated at least two times. **(B)** Fungal burden in the lungs of *Tcra*$^{-/-}$ mice with CnH99 infection only or C-IRIS. *n=6* mice per group. Data are presented as mean values ± SEM.

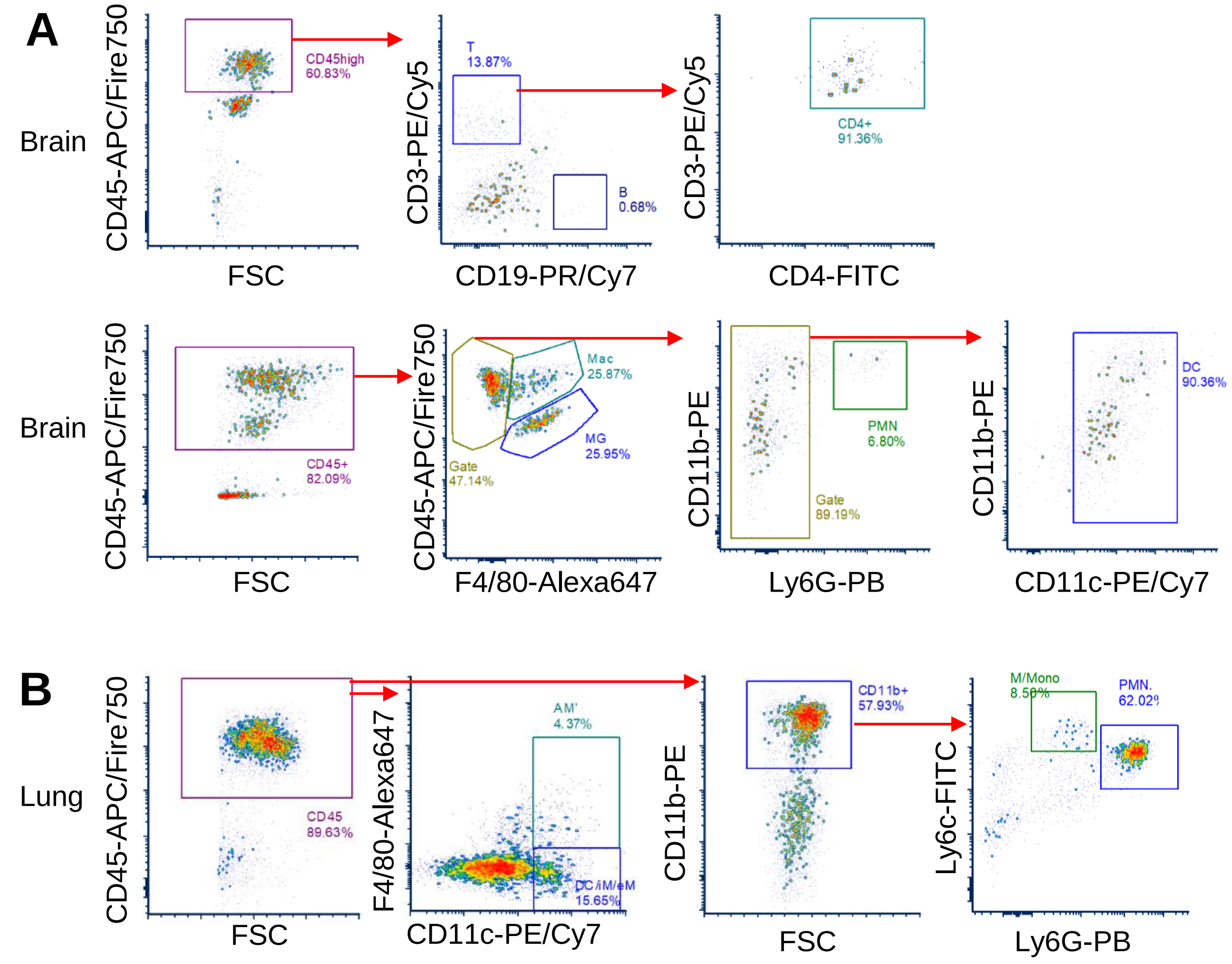

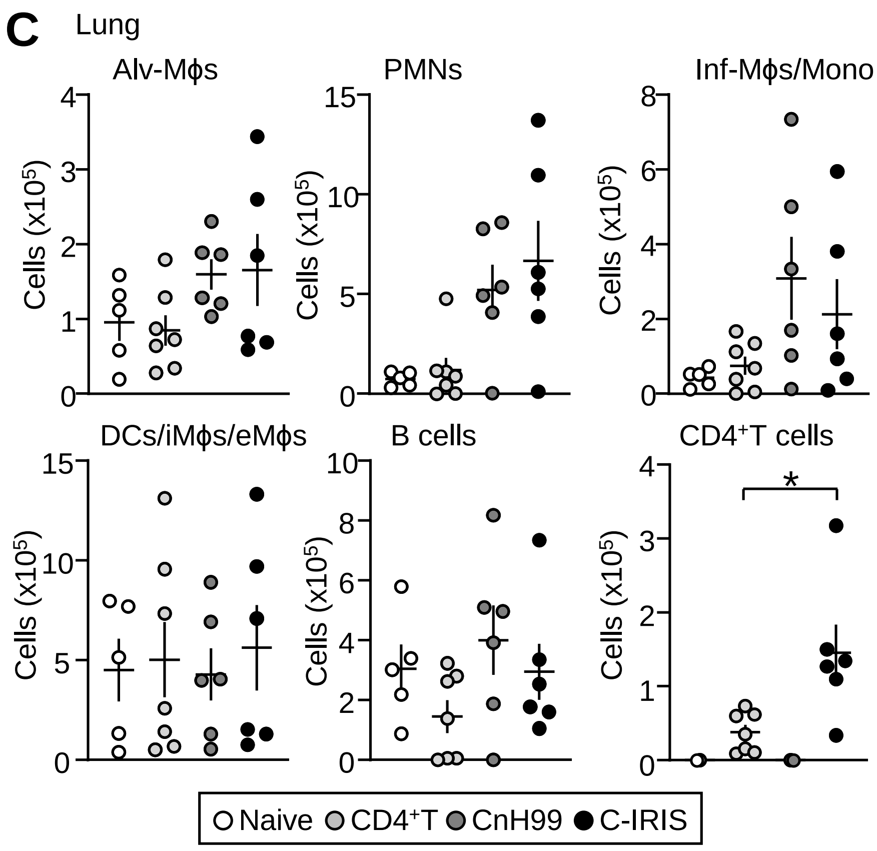

Supplementary Figure 3. Flow cytometry of immune cell distribution in the brains and lungs. (A, B) Gating strategies. **(C)** Cell numbers in the lungs of four cohorts of *Tcra*$^{-/-}$ mice that 1) received neither CnH99 infection nor CD4^{+} T cell transfer, 2) received CD4^{+} T cells for seven days, 3) received CnH99 infection for three weeks + 7 days, and 4) received CnH99 infection for three weeks and then CD4^{+} T cells for seven days. *n=5-7* mice per group. For the CD4^{+} T cell population, a two-tailed unpaired Student's t-test was used between CD4^{+} T cell alone and C-IRIS groups. *: $p < 0.05$. Data are presented as mean values ± SEM.

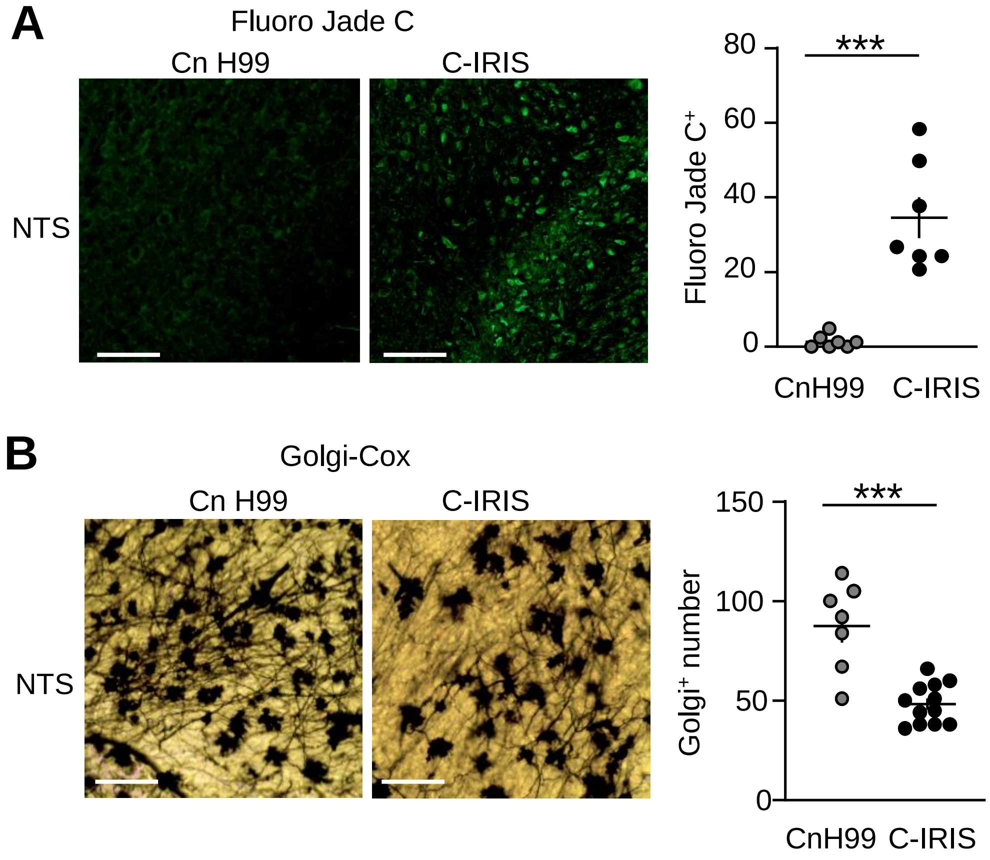

Supplementary Figure 4. Neuronal damage in C-IRIS mice. (A) Fluoro-Jade C staining. *n=7* mice per group. Two-tailed unpaired Student's t-tests were used. Data are presented as mean values ± SEM. ***: $p < 0.001$. **(B)** Golgi-Cox silver staining in the brain of C-IRIS (CnH99+CD4^{+} T) *Tcra*$^{-/-}$ mice. *n=7-11* mice per group. The scale bar indicates 50 μm. Two-tailed unpaired Student's t-tests were used. ***: $p < 0.001$. All data are presented as mean values ± SEM.

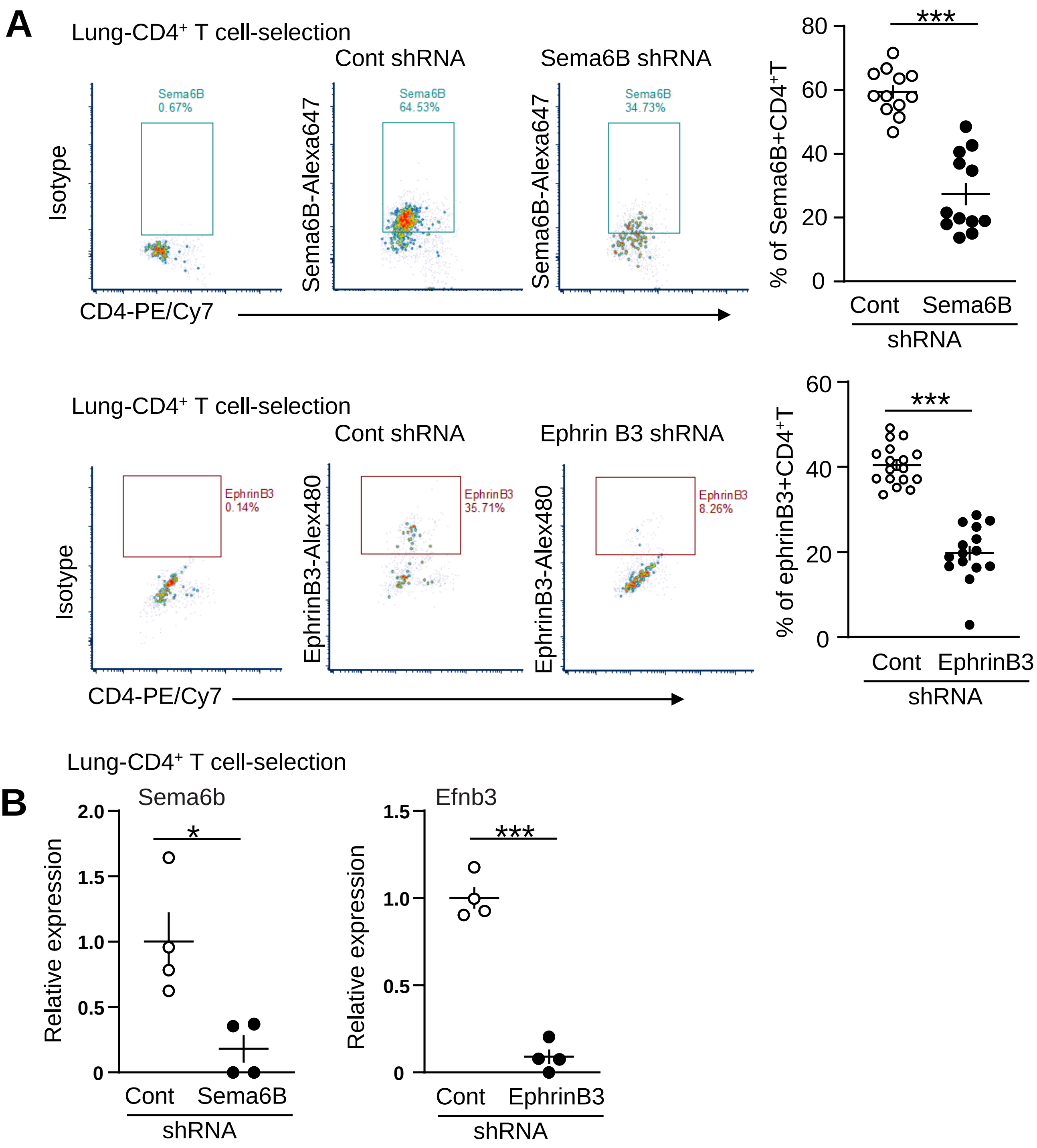

Supplementary Figure 5. Analyses of lung T cell distribution after shRNA treatment. (A) $CD4^+$ T cell selection in the lungs via flow cytometry. Percentages of $Sema6B^+$ or $ephrinB3^+$ $CD4^+$ T cells after control or the respective shRNA treatment. *n=12-17* mice per group. Two-tailed unpaired Student's t-tests were used. ***: $p < 0.001$. **(B)** Relative expression from RT-PCR of Sema6B or ephrinB3 on $CD4^+$ T cells after control or the respective shRNA treatment. *n=4* mice per group. Two-tailed unpaired Student's t-tests were used. *: $p < 0.05$, ***: $p < 0.001$. All data are presented as mean values ± SEM.

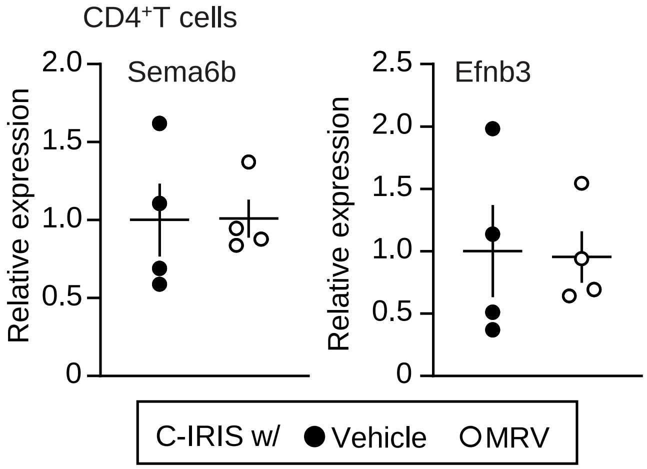

Supplementary Figure 6. Relative expression from RT-PCR of Sema6B or ephrinB3 on CD4$^+$ T cells in C-IRIS mice treated with vehicle or Maraviroc (MRV). CD4$^+$ T cells were isolated from C-IRIS *Tcra*$^{-/-}$ mice with vehicle or MRV treatment (7 days after CD4$^+$ T cells and vehicle/MRV treatment). *n=4* mice per group. Data are presented as mean values ± SEM.

Supplementary Table 1. Primer sequence

Gene	Forward primer	Reverse primer
Sema3a	GGG ACT TCG CTA TCT TCA GAA C	GTC ATC TTC AGG GTT GTC ACT C
Sema3d	ACA TTC CCC ATT CCA GAC AC	TTC GTT GCC CAC CTA CAT C
Sema3f	ACG CTA TGA GGT GCT TTT CC	TGG TCA TGG TCT TAA CAG GTG
Sema4a	GCC GAT TCT CCC TCT GTT TC	GTC TCC TTG TTC AGC TCC TTG
Sema4d	GGG AAA AGT GAA GAT GGC AAA G	CTG TGG GAA GAG TTT CGA GAG
Sema6b	TGT GGT TCG TGT TCC TGT TG	CAT CTT GCT CAA ACG TGG C
Efna1	CAT CTC CAA ACC TAT CTA CCA TCA G	TGC AAA ACC TGT ACT TCC GG
Efna2	CTT TGA GTT CCG GCC TGG	TGT TAC TGG TGA AGA TGG GC
Efna5	GGT GTT CAT GAT CGT GTT TTC G	CTG GGT ATC CTT GGT GTC TG
Efnb1	AAG TTC CAA GAG TTC AGC CC	ATC TTC ATA GTG CGG GTG C
Efnb2	AGA CAA GAG CCA TGA AGA TCC	ACT TCT CCC ATT TGT ACC AGC
Efnb3	CAT AAT TGC CAC ATC AGA CGG	TTC AGA CAC AGG TTT TCG GG
Efnb5	GGT GTT CAT GAT CGT GTT TTC G	CTG GGT ATC CTT GGT GTC TG
Ccr5	TCC AGC AAG ACA ATC CTG ATC	AAC CAT TCC TAC TCC CAA GC
