# Supplementary material for: T cell infiltration into the brain triggers pulmonary dysfunction in murine Cryptococcus-associated IRIS
Source: Nat Commun. 2023 Jun 28;14:3831. doi: 10.1038/s41467-023-39518-x (PMC10307837; doi:10.1038/s41467-023-39518-x)
Supplement: Supplementary file 3 — Reporting Summary [file 41467_2023_39518_MOESM3_ESM.pdf]

nature portfolio

Corresponding author(s): Makoto Inoue

Last updated by author(s): 06/01/2023

Reporting Summary

Nature Portfolio wishes to improve the reproducibility of the work that we publish. This form provides structure for consistency and transparency in reporting. For further information on Nature Portfolio policies, see our Editorial Policies and the Editorial Policy Checklist.

Statistics

For all statistical analyses, confirm that the following items are present in the figure legend, table legend, main text, or Methods section.

n/a	Confirmed	
☐	☒	The exact sample size (*n*) for each experimental group/condition, given as a discrete number and unit of measurement
☐	☒	A statement on whether measurements were taken from distinct samples or whether the same sample was measured repeatedly
☐	☒	The statistical test(s) used AND whether they are one- or two-sided<br>*Only common tests should be described solely by name; describe more complex techniques in the Methods section.*
☒	☐	A description of all covariates tested
☒	☐	A description of any assumptions or corrections, such as tests of normality and adjustment for multiple comparisons
☐	☒	A full description of the statistical parameters including central tendency (e.g. means) or other basic estimates (e.g. regression coefficient) AND variation (e.g. standard deviation) or associated estimates of uncertainty (e.g. confidence intervals)
☐	☒	For null hypothesis testing, the test statistic (e.g. *F*, *t*, *r*) with confidence intervals, effect sizes, degrees of freedom and *P* value noted<br>*Give P values as exact values whenever suitable.*
☒	☐	For Bayesian analysis, information on the choice of priors and Markov chain Monte Carlo settings
☒	☐	For hierarchical and complex designs, identification of the appropriate level for tests and full reporting of outcomes
☒	☐	Estimates of effect sizes (e.g. Cohen's *d*, Pearson's *r*), indicating how they were calculated

Our web collection on statistics for biologists contains articles on many of the points above.

Software and code

Policy information about availability of computer code

Data collection	*Provide a description of all commercial, open source and custom code used to collect the data in this study, specifying the version used OR state that no software was used.*
Data analysis	Whole body plethysmography data were analyzed using Ponemah® Software, Ver 5.2.. Flow cytometry data were analyzed using FCS Express version 6.. Statistical analyses and graphical presentations were computed with GraphPad Prism software (Ver. 9, GraphPad, La Jolla, United States).

For manuscripts utilizing custom algorithms or software that are central to the research but not yet described in published literature, software must be made available to editors and reviewers. We strongly encourage code deposition in a community repository (e.g. GitHub). See the Nature Portfolio guidelines for submitting code & software for further information.

Data

Policy information about availability of data

All manuscripts must include a data availability statement. This statement should provide the following information, where applicable:

- Accession codes, unique identifiers, or web links for publicly available datasets
- A description of any restrictions on data availability
- For clinical datasets or third party data, please ensure that the statement adheres to our policy

All data are provided in the source data file. The atlas used was the Mouse Brain in Stereotaxic Coordinates by Keith B.J. Franklin and George Paxinos.

Human research participants

Policy information about studies involving human research participants and Sex and Gender in Research.

Reporting on sex and gender	N/A
Population characteristics	N/A
Recruitment	N/A
Ethics oversight	N/A

Note that full information on the approval of the study protocol must also be provided in the manuscript.

Field-specific reporting

Please select the one below that is the best fit for your research. If you are not sure, read the appropriate sections before making your selection.

☒ Life sciences ☐ Behavioural & social sciences ☐ Ecological, evolutionary & environmental sciences

For a reference copy of the document with all sections, see nature.com/documents/nr-reporting-summary-flat.pdf

Life sciences study design

All studies must disclose on these points even when the disclosure is negative.

Sample size	No statistical methods were used to predetermine sample sizes, but our sample sizes are similar to those generally employed in the field (Inoue et al., 2016; Khaw et al.,2021).
Data exclusions	No data is excluded from study.
Replication	Experiments were repeated at least two times successfully.
Randomization	Animals were randomly selected in condition allocation process.
Blinding	Investigators were blinded to group allocation during experiment and analysis.

Reporting for specific materials, systems and methods

We require information from authors about some types of materials, experimental systems and methods used in many studies. Here, indicate whether each material, system or method listed is relevant to your study. If you are not sure if a list item applies to your research, read the appropriate section before selecting a response.

Materials & experimental systems

n/a	Involved in the study	
☐	☒	Antibodies
☒	☐	Eukaryotic cell lines
☒	☐	Palaeontology and archaeology
☐	☒	Animals and other organisms
☒	☐	Clinical data
☒	☐	Dual use research of concern

Methods

n/a	Involved in the study	
☒	☐	ChIP-seq
☐	☒	Flow cytometry
☒	☐	MRI-based neuroimaging

Antibodies

Antibodies used	rabbit anti-mouse beta-3 tubulin antibody (Novus Biologicals, Cat# NBP130048; 1:200). rabbit c-Fos primary antibody (1:500, Cell Signaling Cat# 2250). CD19 (Cat# 115504, BioLegend), CD11c (Cat# 117304, BioLegend), CD8 (Cat# 100704, BioLegend), Ly6G (Cat# 127604, BioLegend), and CD11b (Cat# 101204, BioLegend).
Validation	rabbit anti-mouse beta-3 tubulin antibody (Novus Biologicals, Cat# NBP130048; 1:200): This antibody has been validated by Biological Strategies.

	rabbit c-Fos primary antibody (1:500, Cell Signaling Cat# 2250): This antibody has been validated using SimpleChIP® Enzymatic Chromatin IP Kits.<br>Flow antibodies have been validated by BioLegend.

Animals and other research organisms

Policy information about studies involving animals; ARRIVE guidelines recommended for reporting animal research, and Sex and Gender in Research

Laboratory animals	C57BL6, Rag1-/- (002216), Tcra-/- (002116), CCR5-/- (005427), Slc17a7-IRES2-Cre knock-in mice (Vglut1-cre, 023527), CD4-cre (022071), and floxed EYFP (7903) mice were used in the study. Mice were group-housed with two to five in an individual cage and kept under specific pathogen-free conditions with a 12-h light/dark cycle. Sterile pelleted mouse diet and water were given for the health monitoring of mice.
Wild animals	No wild animals were used.
Reporting on sex	Healthy male and female mice were used in the study.
Field-collected samples	There are no field collected samples.
Ethics oversight	All studies and experiments were performed under the approval of the Institutional Animal Care and Use Committee at the University of Illinois.

Note that full information on the approval of the study protocol must also be provided in the manuscript.

Flow Cytometry

Plots

Confirm that:

☒ The axis labels state the marker and fluorochrome used (e.g. CD4-FITC).

☒ The axis scales are clearly visible. Include numbers along axes only for bottom left plot of group (a 'group' is an analysis of identical markers).

☒ All plots are contour plots with outliers or pseudocolor plots.

☒ A numerical value for number of cells or percentage (with statistics) is provided.

Methodology

Sample preparation	Seven days after CD4+ T cell transfer, brains and lungs were retrieved. Tissues were excised and minced in PBS supplemented with collagenase D (1 mg/ml). Minced tissues were incubated for 30 minutes at 37°C, filtered through the 80-µm mesh, and then centrifuged at 277 g at 4C. To isolate mononuclear cells from the brains and lungs, cells were resuspended in 30% Percoll (in PBS), laid over 70 % Percoll, and centrifuged at 377 g for 20 minutes at room temperature. Before cell staining with antibodies, Fc receptors were blocked with Fc Block (BD Pharmingen) for 7 min on ice. Cells were stained with fluorochrome-conjugated specific antibodies (1:200) for 30 mins on ice, followed by washing and collecting cells.
Instrument	Stained cells were analyzed on Cytek Aurora with the FCS Express (De Novo).
Software	Data were analyzed using FCS Express version 6.
Cell population abundance	Cell population abundance were used according to previous report (Khaw et al., Frontier in Immunology, 2020)
Gating strategy	In the lung samples, DC or interstitial or exudate macrophages (DC/iMΦ/eMΦ) were identified as CD45+CD11c+F4/80-, alveolar macrophages (AM) as CD45+CD11c+F4/80+, inflammatory macrophage/monocyte as CD45+Ly6C+CD11b+, neutrophils as CD45+Ly6GhiCD11b+, and CD4+ T cells as CD45+CD3+CD4+. In the brain samples, CD4+ T cells were identified as CD45+CD3+CD4+, microglia as CD45loF4/80+, macrophage as CD45hiF4/80+, and neutrophils as CD45+Ly6G+. These gating strategy were used acording to previous report (Khaw et al., Frontier in Immunology, 2020)

☒ Tick this box to confirm that a figure exemplifying the gating strategy is provided in the Supplementary Information.
